# Combined LA-ICP-MS/LIBS: powerful analytical tools for the investigation of polymer alteration after treatment under corrosive conditions

**DOI:** 10.1038/s41598-020-69210-9

**Published:** 2020-07-27

**Authors:** Lukas Brunnbauer, Maximilian Mayr, Silvia Larisegger, Michael Nelhiebel, Laura Pagnin, Rita Wiesinger, Manfred Schreiner, Andreas Limbeck

**Affiliations:** 1 Institute of Chemical Technologies and Analytics, TU Wien, Getreidemarkt 9/164-IAC, 1060 Vienna, Austria; 2 KAI Kompetenzzentrum Automobil- und Industrieelektronik GmbH, Technologiepark Villach Europastraße 8, 9524 Villach, Austria; 3 Institute of Science and Technology in Art, Academy of Fine Arts Vienna, Schillerplatz 3, 1010 Vienna, Austria

**Keywords:** Analytical chemistry, Characterization and analytical techniques, Polymers

## Abstract

Polymers are used in a variety of different areas, including applications in food packaging, automotive and the semiconductor industry. Information about degradation of these materials during application, but also uptake of pollutants from the surrounding environment is therefore of great interest. Conventional techniques used for polymer characterization such as FT-IR or Raman spectroscopy, but also thermo-analytical techniques offer insights into degradation processes but lack the possibility to detect uptake of inorganic species. Moreover, these techniques do not allow the measurement of depth profiles, thus information about degradation or pollutant uptake with sample depth is not accessible. In this work, we propose LA-ICP-MS and LIBS as powerful analytical tools for polymer characterization, overcoming the limitations of conventional analytical techniques used for polymer analysis. Applicability of the developed procedures is demonstrated by the analysis of artificially weathered polyimides and modern art materials, indicating that the degradation of the polymer but also the uptake of corrosive gases is not limited to the sample surface. Finally, a tandem LA-ICP-MS/LIBS approach is employed, which combines the advantages of both laser-based procedures, enabling the simultaneous analysis of polymer degradation and cadmium uptake of polystyrene after exposure to UV radiation and treatment with artificial sea water.

## Introduction

Synthetic polymers and plastics are among the most commonly used materials in our modern world^1^. They are mainly employed as packaging materials for consumer goods such as food and cosmetics and also bottles and boxes, but also frequently applied for construction materials^2^. Polymers are also used as passivation or encapsulation materials in the semiconductor industry^3,4^ or, in combination with pigments, as paints in the fields of art and cultural heritage^5,6^. In general, the applied synthetic polymers are composed of an organic-carbon-chain polymer and different additives that give the materials the intended chemical and physical properties. Commonly applied additives include plasticizers, antioxidants, antistatic agents, lubricants, flame retardants or inorganic pigments.

During application, polymers are often exposed to harmful environmental conditions, causing changes in their chemical composition. In this context, the negative influences of sunlight but also contact with ambient gases and environmental liquids have to be mentioned. Whereas UV light and oxidative gases are known to promote degradation^7–9^, corrosive gases or metals dissolved in rain, snow and river or sea-water are susceptible for uptake into the polymer network, resulting in increased concentrations of inorganic constituents in aged materials. All of these possible interactions contribute to unwanted changes in the polymer composition, which finally lead to altered material properties (e.g. bleaching of colors, reduced thermal stability, increased brittleness, etc.). A comprehensive characterization of aged polymers is therefore necessary not only to control if the polymers still fulfill the requirements for further application (e.g. in the semiconductor industry^10–14^), but also to achieve desired material properties (e.g. resistance to weathering is important in the field of cultural heritage research^15–17^).

At the end of their life-cycle, polymers often end up in the environment, for example in the form of microplastics which pose a significant threat to various ecosystems^18^. Accordingly, the composition and metal contents of the degraded polymers should be monitored, to better estimate the adverse health effects of microplastics in the environment^19–21^.

Due to their wide range of properties and applications, the composition of synthetic polymers is rather versatile. Polymer analysis is therefore a great challenge to analytical chemistry. The most commonly used analytical techniques for characterization and analysis of polymers are FT-IR and Raman spectroscopy. Besides being able to identify and classify different polymers, these techniques are also employed to investigate polymer degradation^22–25^. Furthermore, Py-GC–MS^26^ and MALDI-ToF–MS^27^ are also commonly applied for polymer analysis, providing information about the molecular composition of the investigated polymer sample. Thermogravimetric analysis (TGA) and differential thermal analysis (DTA) are frequently used techniques giving bulk information about thermo-oxidative polymer degradation^22,28,29^. However, information about the metal contents prevailing in the polymer samples is not accessible with these techniques. For this purpose, polymers are first converted into a solution and subsequently analyzed using liquid analysis techniques such as inductively coupled plasma-optical emission spectroscopy (ICP-OES) or inductively coupled plasma-mass spectrometry (ICP-MS)^30,31^. However, the applied digestion methods are always accompanied by the risk of contamination or elemental loss and the complete procedure of analysis is often time-consuming. Nowadays, elemental analysis of polymers is carried out using solid sampling techniques such as electrothermal vaporization [^32,33^] or laser ablation^34,35^ in combination with ICP-OES or ICP-MS detection to overcome the limitations of wet chemical analysis. With these techniques, accurate and highly sensitive trace elemental measurements are possible. However, they do not offer information about polymer degradation. Summing up, with the analytical procedures reported so far it is not possible to study polymer degradation and inorganic species with one single measurement.

In this work, we present an analytical technique that permits the simultaneous detection of polymer degradation as well as changes in the elemental composition. The developed procedure is based on the concurrent sample analysis using laser induced breakdown spectroscopy (LIBS) and laser ablation-inductively coupled plasma-mass spectrometry (LA-ICP-MS). In this so-called tandem LA-ICP-MS/LIBS approach a focused laser beam is fired on the sample surface. With LIBS, the radiation emitted by the formed plasma plume is detected. Thereby information about the major components of polymers, namely carbon, oxygen, hydrogen and nitrogen but also molecular sample information in the form of the C_2_ swan band and the CN violet band is collected^36^. Together with different statistical methods, the acquired LIBS data can be used for compound identification. Broadband LIBS spectra have, for example, already been used for the classification of different polymer types^37,38^. The aerosol generated in the ablation process is measured with LA-ICP-MS and provides data about the inorganic sample constituents^39^. Attributes making LA-ICP-MS attractive for the analysis of trace elements in solid samples are the high sensitivity, reaching LODs in the range of μg·g^−1^ to ng·g^−1^, the large dynamic working range and the capability for multi-elemental analysis^40,41^. The combination of these laser based analytical techniques allows overcoming the above stated limitations of state-of-the-art polymer characterization techniques. Furthermore, the proposed procedure does not only allow the analysis of surface near sample regions, the ability to measure depth profiles provides also information about the distribution within the sample. The advantages of this tandem LA-ICP-MS/LIBS setup are demonstrated by the analysis of polymer samples from the fields of the semiconductor industry, cultural heritage science and environmental research. Derived findings are discussed in detail in the context of this work.

## Experimental

### Instrumentation and sample analysis

LA-ICP-MS measurements were carried out using an ESI NWR213 (Fremont, CA) laser ablation system operating at a wavelength of 213 nm coupled to an iCAP Qc ICP-MS system (ThermoFisher Scientific, Bremen, Germany) using PTFE tubing. The samples were ablated under a constant stream of helium (0.65 L/min). Argon was used as a make-up gas (1 L/min) before introducing the aerosol to the ICP-MS. Tuning of the instrument was carried out daily for maximum ^115^In signal using a NIST612 glass standard (National Institute of Standards and Technology, Gaithersburg, MD). Data was collected using Qtegra 2.10 provided by the manufacturer of the instrument.

LIBS experiments were carried out using a commercially available LIBS J200 system (Applied Spectra, Inc., Fremont, CA). A frequency quadrupled Nd:YAG laser operating at a wavelength of 266 nm with a 5 ns pulse duration was used for ablation and excitation. Emitted radiation after each laser pulse was collected using two different collection optics connected to optical fibers: one collection optic optimized for UV-light (188–300 nm) and a second collection optic for the remaining part of the spectrum (300–1.048 nm). The collected light was analyzed using a Czerny-Turner spectrometer with 6 channels covering a total wavelength range from 188 to 1.048 nm. LIBS data was recorded using Axiom 2.0 software provided by the manufacturer of the instrument.

Tandem LA-ICP-MS/LIBS measurements were carried out by directly coupling the ablation chamber of the LIBS J200 system to the ICP-MS using PTFE tubing with a 3 mm inner diameter and a length of 1.2 m. He (0.6 L/min) was used as a carrier gas which was mixed with Ar (0.6 L/min) using a t-piece placed directly after the ablation chamber. This setup allows the simultaneous acquisition of LIBS, as well as ICP-MS data, increasing the total information obtained from each measurement.

For analysis using LA-ICP-MS, LIBS or Tandem LA-ICP-MS/LIBS the polymer samples of interest were fixed on high purity silicon wafers (Infineon Austria AG, Villach, Austria). The applied measurement parameters are described in detail in the respective results sections.

Crater depths obtained in depth profile measurements as well as coating thicknesses were determined using a profilometer (DektakXT, Bruker, Massachusetts, USA).

### Reagents

30% (v/v) H_2_O_2_ (p.a.) supplied by Merck (Darmstadt, Germany) was used for aging experiments of polystyrene samples. Artificial seawater (prepared as described by Kester et al.^43^) spiked with 10 ppb of Cd (ICP-multi elemental solution VIII in diluted HNO_3_ obtained from Sigma–Aldrich, Buchs, Switzerland) was used for exposure of polystyrene samples. Metal-free water (resistivity 18.2 MΩ cm^−1^) dispensed from a Barnstead EASYPURE II water system (ThermoFisher Scientific, Marietta) was used for sample rinsing and dilutions. H_2_S (100 ppm), SO_2_ (100 ppm) and synthetic air (5.0) used for weathering experiments was supplied by Messer, Austria. O_3_ was produced using an ozonisator (Airmaster OMX 500, Topchem GmbH, Germany) with O_2_ (5.0) supplied by Messer, Austria.

### Weathering equipment

Accelerated stress tests were performed to cause degradation of the polymers as well as uptake of sulfur within the investigated samples. Therefore, samples were exposed to synthetic air in combination with corrosive gases (SO_2_, H_2_S and O_3_) or UV light in two separate chambers. For weathering experiments with corrosive gases, a chamber (Bel-Art, SP Scienceware) with gas in‐ and outlets with a total volume of 30 cm^3^ was used. To generate the desired concentration of corrosive gases, synthetic air is humidified using double-distilled water and mixed with the different gases. The chamber is continuously flushed with the gas mixture with a gas flowrate of 100 L/h. A detailed description of the weathering chamber is given by Wiesinger et al.^42^. For simulating artificial sunlight, an UVACUBE chamber (Dr Hönle GmbH, Germany) equipped with a xenon arc lamp with 170 W/m^2^ was used.

Note that the detailed weathering procedure for each example of application (i.e. polyimide samples, paint samples and polystyrene samples) is described in the respective result and discussion sections.

### Sample preparation

To demonstrate the broad application range of laser ablation techniques, three different polymer samples are investigated within this work.

Paint samples relevant in the field of cultural heritage science were prepared by casting a gravimetric 1:3 mixture of an inorganic pigment (manganese violet (NH_4_MnP_2_O_7_, Kremer Pigmente, Germany) and an organic polymeric binder (Alkyd, Lukas Farben, Germany) on a glass slide. A total wet thickness of 150 µm was achieved. After preparation, the samples were dried for one week under ambient conditions, resulting in a final thickness of 100 µm. Note that these samples are referred to as paint samples from now on.

Standard polyimide samples (6 µm thickness) were provided by Infineon Technologies (Infineon Austria AG, Villach, Austria). Samples were cut into 10 × 10 mm^2^ pieces. Note that these samples are referred to as polyimide samples from now on.

Polystyrene (PS) films with a thickness of 20 µm were obtained from Goodfellow Inc. (Hamburg, Germany)**.** Samples were manually cut into 10 × 10 mm^2^ pieces using ceramic scissors. Note that these samples are referred to as polystyrene samples from now on.

## Results and discussion

For a complete characterization of aged polymers, information about the progress of polymer degradation but also about the absorption of pollutants from the environment is required. As reported in several studies, LIBS is a promising tool for the detection of degradation^44,45^, whereas for the second task LA-ICP-MS has been applied^34,35^. However, in all of these works only bulk analyses have been performed. Within this study, polymer degradation and uptake of inorganic species will be analyzed as a function of the sample depth for the first time. In a first step the ability of LIBS and LA-ICP-MS to measure depth profiles has been exploited. Finally, the two analytical techniques were combined in a tandem LA-ICP-MS/LIBS approach.

### Depth profiling of polymer degradation and oxidation using LIBS

In this section, a LIBS method is developed that allows detection of polymer degradation as well as oxidation of the sample. The developed method is applied to perform depth profile investigations of aged paint samples.

As LIBS has already been used for (spatially resolved) polymer classification^36,46,47^, it is a promising tool for the dectection of polymer degradation. LIBS inherently enables analysis of oxygen and should therefore also be able to detect oxidation of polymers. Additionally, other polymer specific LIBS signals may change due to degradation and aging of the sample, enabling measurement of polymer degradation.

**Figure 1:**
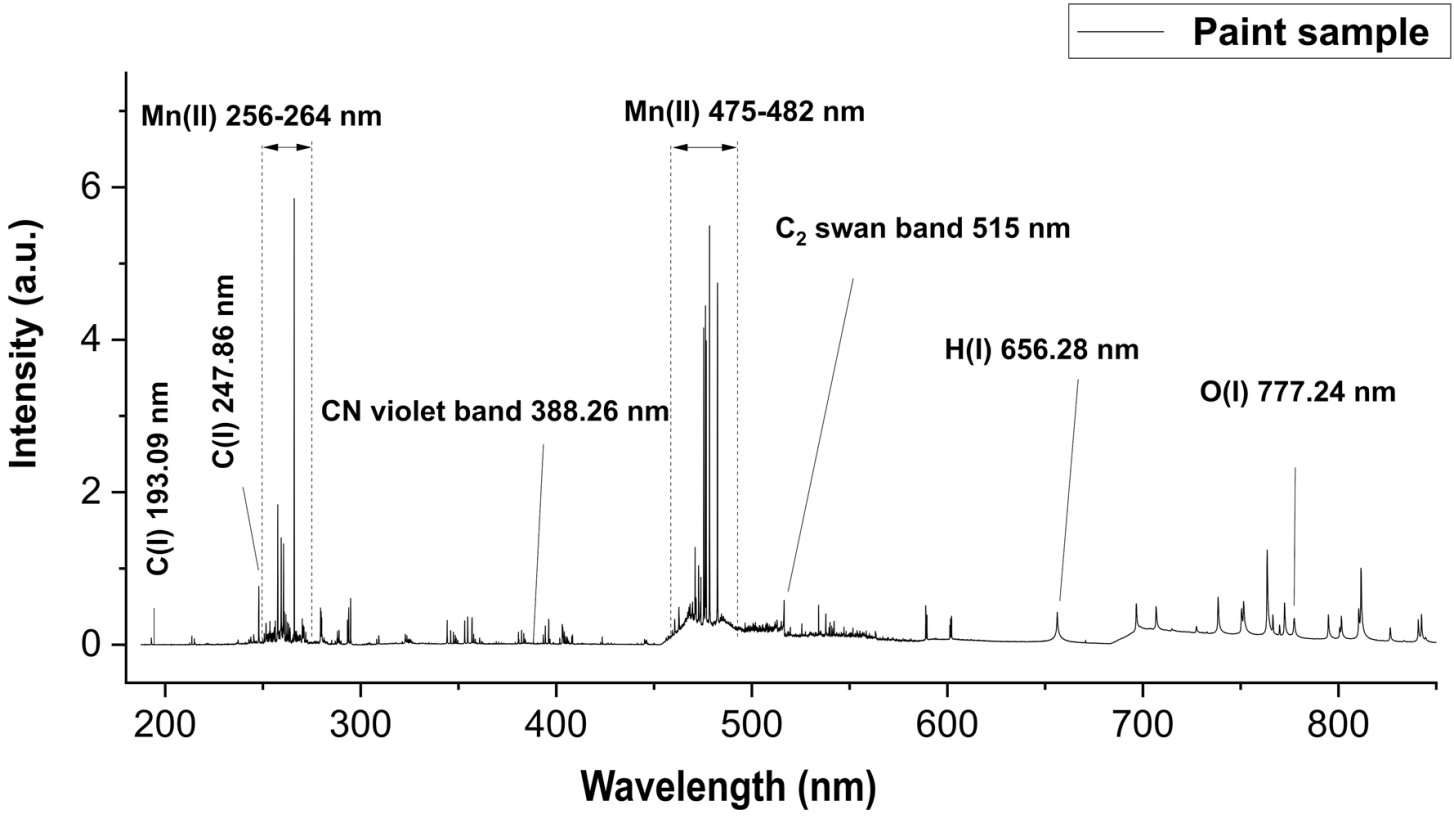
Representative LIBS spectrum of the investigated paint samples. Aside from polymer specific emission signals, signals from the inorganic pigment (manganese violet) are observed.

Figure 1 shows a representative LIBS spectrum of the investigated unaged paint sample consisting of an inorganic pigment (manganese violet) and a polymeric binder (alkyd). Emission signals of both materials are found and marked in the representative LIBS spectrum.

In a preliminary set of experiments laser energy and gate delay were optimized for a high signal-to-noise ratio in the regions of interest of the LIBS spectrum. Emission signals of polymers observed in LIBS spectra usually consist of the atomic emission lines of the main components carbon, hydrogen, oxygen and nitrogen, where short gate delays yield higher signal-to-noise ratios. In addition, molecular emission signals (e.g. C_2_ swan band) are observed, which are reported to appear sooner in the plasma and consequently require a short gate delay too^48^. Table 1 shows the optimized LIBS parameters.

**Table 1:** LIBS measurement parameters for paint samples.

**Laser system (Applied Spectra J200)**	
Laser fluence (J/cm^2^)	29.03
Laser spotsize (µm)	100
Laser repetition rate (Hz)	10
Laser beam geometry	Circular
Stage scan-speed (mm/s)	1
Atmosphere	Argon
Laser wavelength (nm)	266
**Spectrometer system (Czerny-Turner)**	
Detection channels	6
Gate delay (µs)	0.3
Gate width (ms)	1.05
Covered wavelength range (nm)	188–1048

LIBS measurements were carried out using patterns of 10 parallel line scans with a distance of 100 µm between each line and a total length of 2 mm each, resulting in 21 shots per line and 210 recorded spectra per pattern. For data evaluation, obtained LIBS spectra of each layer of each sample were averaged and the standard deviation was calculated. LIBS signals of interest were integrated and background correction was performed using the mean value of 5 neighboring pixels of the detector when integrating each emission signal.

Weathering parameters were especially focused on conditions conventionally used in the field of heritage science^49–51^. Paint samples with a thickness of 100 µm were weathered under two different conditions: To determine the degradation of polymers, one set of paint samples was exposed to UV radiation for 1 up to 6 weeks. To investigate the oxidation of polymers a second set of paint samples was weathered with a gas mixture containing 10 ppm O_3_ (20 mg/m^3^) and 10 ppm SO_2_ (26 mg/m^3^) and 80% relative humidity in synthetic air for 72 h.

**Figure 2:**
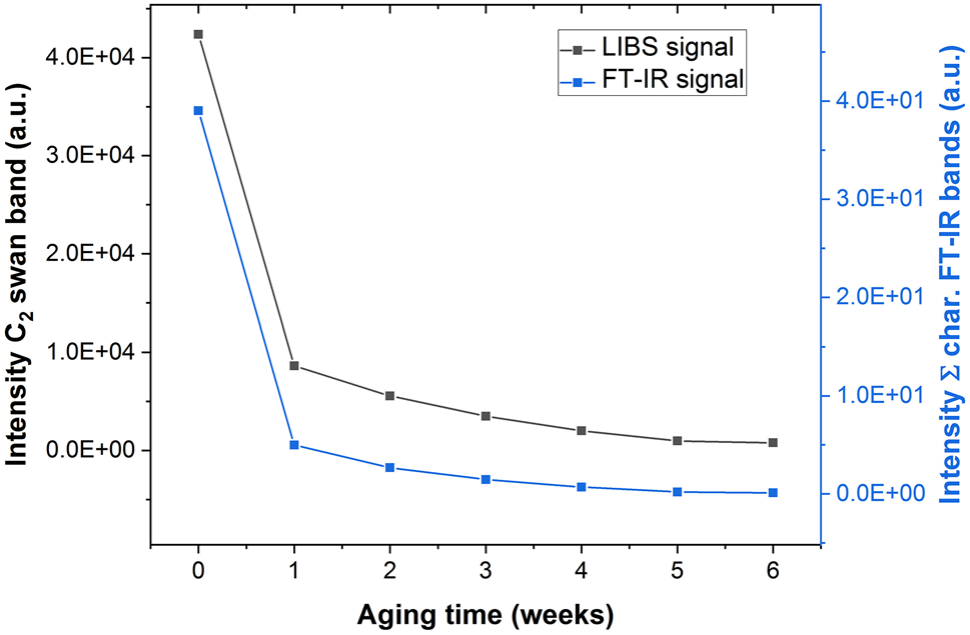
Trend of polymer degradation on the sample surface of paint samples aged for 1 up to 6 weeks detected with FT-IR (sum of C-H stretch at 2930 cm^-1^, C=O stretch at 1730 cm^−1^, and C–O stretch at 1100 cm^−1^) and detected using the C_2_ swan band from LIBS measurements.

Paint samples exposed to UV radiation for 1 up to 6 weeks were used to evaluate the applicability of LIBS to detect polymer degradation. The surface of these samples was analyzed using FT-IR spectroscopy (LUMOS, MCT detector, BRUKER Optik GmbH, Germany) and in accordance to literature^51–53^ the main characteristic polymer absorbance bands (C–H stretch at 2930 cm^-1^, C=O stretch at 1730 cm^-1^, and C-O stretch at 1100 cm^-1^) were used to evaluate polymer degradation over aging time. Figure 2 shows the sum of the characteristic polymer FT-IR signal over the aging time of 6 weeks. A significant decrease of the FT-IR signal is observed for increased aging time. The same samples were also analyzed using the developed LIBS procedure. For data interpretation the C_2_ swan band intensity, derived via accumulation of the 210 recorded spectra per sample pattern, is plotted against the aging time (Fig. 2). As the trend of obtained LIBS results is in good agreement with the trend of FT-IR measurements, it is confirmed that LIBS can be used to detect polymer degradation.

**Figure 3:**
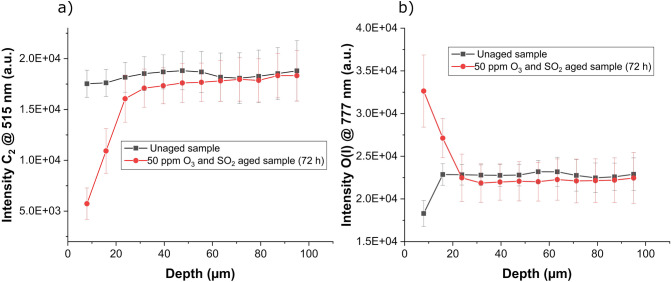
Depth profile measurement comparing a sample exposed to a mixture of 50 ppm O_3_ and 50 ppm SO_2_ for 72 h to an unaged sample: (**a**) Changes in the C_2_ swan band are used to detect polymer degradation and (**b**) O emission signal indicates oxidation of the investigated paint sample. Average signals and standard deviations are calculated from 210 LIBS spectra recorded per layer.

In a next step, depth profiles of paint samples exposed to a mixture of 50 ppm O_3_ (38 mg/m^3^) and SO_2_ (131 mg/m^3^) for 72 h are analyzed using LIBS. For depth profile measurements the applied multiple line patterns were repeated 12 times at the same position, resulting in a total depth of 95 µm and a thickness of approximately 6.7 µm per layer. Using the C_2_ swan band, it is now possible to investigate the propagation of the degradation into the sample. Depth profile measurements comparing the aged sample to an unaged sample are shown in Fig. 3a. Similar as observed for the sample aged with UV light, sample treatment with ozone results in decreased signal intensities for the C_2_ swan band in the degraded sample region. Depth profiles reveal that degradation of the sample did not only occur on the sample surface, even for the second layer-corresponding to a depth of approximately 15 µm—significant differences compared to the reference samples was observed. In layer 3 the average intensity of the C_2_ swan band seems still to be lower for the weathered sample, but this difference disappeared with increasing sample depths. However, weathering in the presence of ozone results not only in sample degradation, there is also the possibility of substantial sample oxidation. Thus, exposure to oxidizing gases should result in increased oxygen contents for the weathered samples. This is also confirmed using LIBS depth profiling. In Fig. 3b the findings for the oxygen emission intensities of aged and unaged reference samples are presented. Compared to the unaged sample a significant increase in the oxygen content was detected in the first two layers (approximately 15 µm) of the aged sample. This outcome is in good agreement with the findings of the C_2_ swan band, indicating that the applied corrosive gases penetrated the first 20 µm of the investigated modern art material. Therefore, LIBS offers not only the possibility to analyse polymer degradation in depth but can also be employed to detect an increase of oxygen caused by oxidation within the sample. These results confirm that LIBS offers many benefits when it comes to analysis of polymer degradation.

### Depth profiling of uptake of sulfur species in polymers using LA-ICP-MS

LA-ICP-MS is an analytical technique commonly reported in the literature for elemental depth profiling experiments^54,55^ but has, to our knowledge, never been employed to investigate the uptake of corrosive species in polymers. In this section, we present LA-ICP-MS as a powerful analytical tool to investigate sulfur uptake within polyimides after weathering experiments. Investigations were focused on the impact of sulfur-dioxide and hydrogen-sulfide as these gases are more prominent in corrosion studies in semiconductor devices^56–58^. In a first set of experiments polyimide samples were exposed to 50 ppm H_2_S (70 mg/m^3^) with 80% relative humidity in synthetic air for 192 h. The second weathering experiment was conducted using 50 ppm SO_2_ (131 mg/m^3^) with 80% relative humidity in synthetic air for 192 h.

The uptake of these corrosive gases was measured using LA-ICP-MS. Measurement parameters were optimized carefully in preliminary experiments for maximum depth resolution while maintaining a high sensitivity for sulfur (Table 2). As the main isotopes of sulfur and silicon ^32^S and ^28^Si are interfered by ^16^O^16^O and ^14^N^14^N respectively, elements which are both present in the investigated sample, the less abundant isotopes ^34^S and ^29^Si were selected for ICP-MS anaylsis.

**Table 2:** LA-ICP-MS measurement parameters for polyimide samples.

**LA system (NWR 213)**	
Laser fluence (J/cm^2^)	1.08
Laser spotsize (µm)	200
Laser repetition rate (Hz)	20
Laser beam geometry	Circular
Stage scan-speed (mm/s)	1
Atmosphere	He
Laser wavelength (nm)	213
**ICP-MS (Thermo iCAP Q)**	
Aux. gas flow (L/min)	0.8
Cool gas flow (L/min)	13
Dwell time per isotope (ms)	10
RF power (W)	1550
Cones	Ni
Measured isotopes	^13^C^+^, ^29^Si^+^, ^34^S^+^

**Figure 4:**
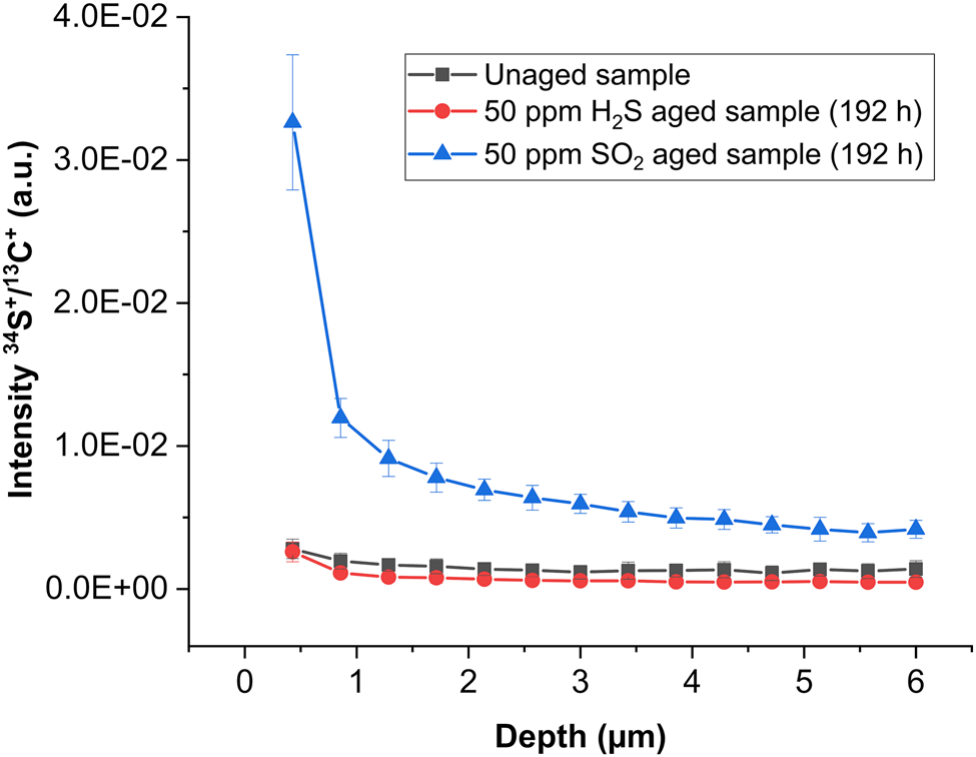
LA-ICP-MS depth profiles of the investigated polyimide sample showing different sulfur uptake depending on the gas used for weathering. Average signals and standard deviations are calculated from three consecutive measurements on the same sample.

Depth profiles of the weathered polyimide samples were carried out by measuring subsequent line scans with a total length of 6 mm repeatedly on the same position for 14 times. Considering a mean ablation rate of around 420 nm per measured line this results in full penetration of the 6 µm tick polyimide film after the last line (indicated by an increase of ^29^Si^+^ signal). Obtained transient signals were averaged and used for further data evaluation. Averaged ^34^S^+^ signals were normalized to ^13^C^+^ to compensate for instrumental drifts during the measurement. Even though this approach is discussed controversially for quantitative measurements in the field of elemental bio-imaging^59^, we believe that it can be used for the correction of instrumental drifts in this work. Especially when considering that the investigated polymer represents a rather homogeneous composition compared to biological tissue samples consisting of various carbonaceous species. Average signals and standard deviations are calculated from three consecutive measurements on the same sample. Depth profiles of sulfur uptake of the investigated polyimide samples exposed to H_2_S, SO_2_ and an unaged reference sample are shown in Fig. 4.

Between the polyimide sample exposed to H_2_S and the unaged reference sample, no significant differences in the ^34^S^+^/^13^C^+^ signal ratios were found, whereas weathering the sample with SO_2_ results in a significant uptake of sulfur. Moreover, it was shown that the uptake is enhanced for surface near regions, and decreases with sample depth. Nevertheless, the substantial sulfur signal measured in the polyimide film near the silicon indicates a complete penetration of the polymer by the gas and thus insufficient protection of the underlying material. Obtained results demonstrate the high potential of LA-ICP-MS for the investigation of uptake of inorganic species in polymers.

### Tandem LA-ICP-MS/LIBS analysis: combining depth profiling of oxidation and trace metal uptake

Results presented in the previous sections demonstrate that LA-ICP-MS/LIBS offer many advantages in terms of polymer analysis compared to conventional analytical techniques, in particular because the spatially resolved analysis of polymer degradation and uptake of contaminants is possible. As both techniques enable investigations with unique features (e.g. high sensitivity for inorganic species, capability to detect degradation and increase of oxygen), combining both techniques in a tandem LA-ICP-MS/LIBS setup should enable a more complete characterization of polymeric samples.

Development of a tandem LA-ICP-MS/LIBS approach, where polymer specific LIBS signals as well as signals from inorganic species are acquired simultaneously, is presented in the following section. Therefore, polystyrene films which served as a synthetic substitute for microplastics were aged using UV radiation and H_2_O_2_. Afterwards the films were exposed to cadmium dissolved in artificial sea water. A tandem LA-ICP-MS/LIBS system was employed to simultaneously investigate the polymer oxidation caused by UV and H_2_O_2_ aging as well as the uptake of cadmium.

For tandem LA-ICP-MS/LIBS measurements, laser parameters have to be optimized taking into account the higher laser fluence typically used for LIBS measurements. Therefore, the laser energy was optimized in preliminary experiments to obtain good depth resolution while maintaining adequate LIBS signal intensity. Tandem LA-ICP-MS/LIBS measurement parameters are shown in Table 3. Depth profiles were recorded by subsequent ablation of line scans with a total length of 2 mm. Penetration of the 20 µm thick samples was achieved after 6 ablated line scans resulting in a depth resolution of 3.3 µm.

**Table 3:** Tandem LA-ICP-MS/LIBS measurement parameters for polystyrene.

**LIBS system (J200)**	
Laser fluence (J/cm^2^)	8.91
Laser spotsize (µm)	100
Laser repetition rate (Hz)	10
Laser beam geometry	circular
Stage scan-speed (mm/s)	1
Atmosphere	He
Laser wavelength (nm)	266
Detection channels	6
Gate delay (µs)	0.3
Gate width (ms)	1.05
Covered wavelength range (nm)	188–1048
**ICP-MS (Thermo iCAP Q)**	
Aux. gas flow (L/min)	0.8
Cool gas flow (L/min)	13
Dwell time per isotope (ms)	10
RF power (W)	1550
Cones	Ni
Measured isotopes	^13^C^+^, ^114^Cd^+^

One side of the polystyrene films was aged for 4 weeks by the combined exposure to UV radiation and 30% (v/v) H_2_O_2_ H_2_O_2_ was repeatedly reapplied to the sample surface to avoid complete evaporation of the liquid. Subsequently, aged samples and unaged blank samples were submerged and exposed from both sides to artificial seawater spiked with 10 ppb of Cd After an exposure time of 24 h, samples were separated from the solution and rinsed with metal-free water to remove remaining droplets of artificial sea water. After a drying step with synthetic air the samples were measured using the developed Tandem LA-ICP-MS/LIBS procedure.

**Figure 5:**
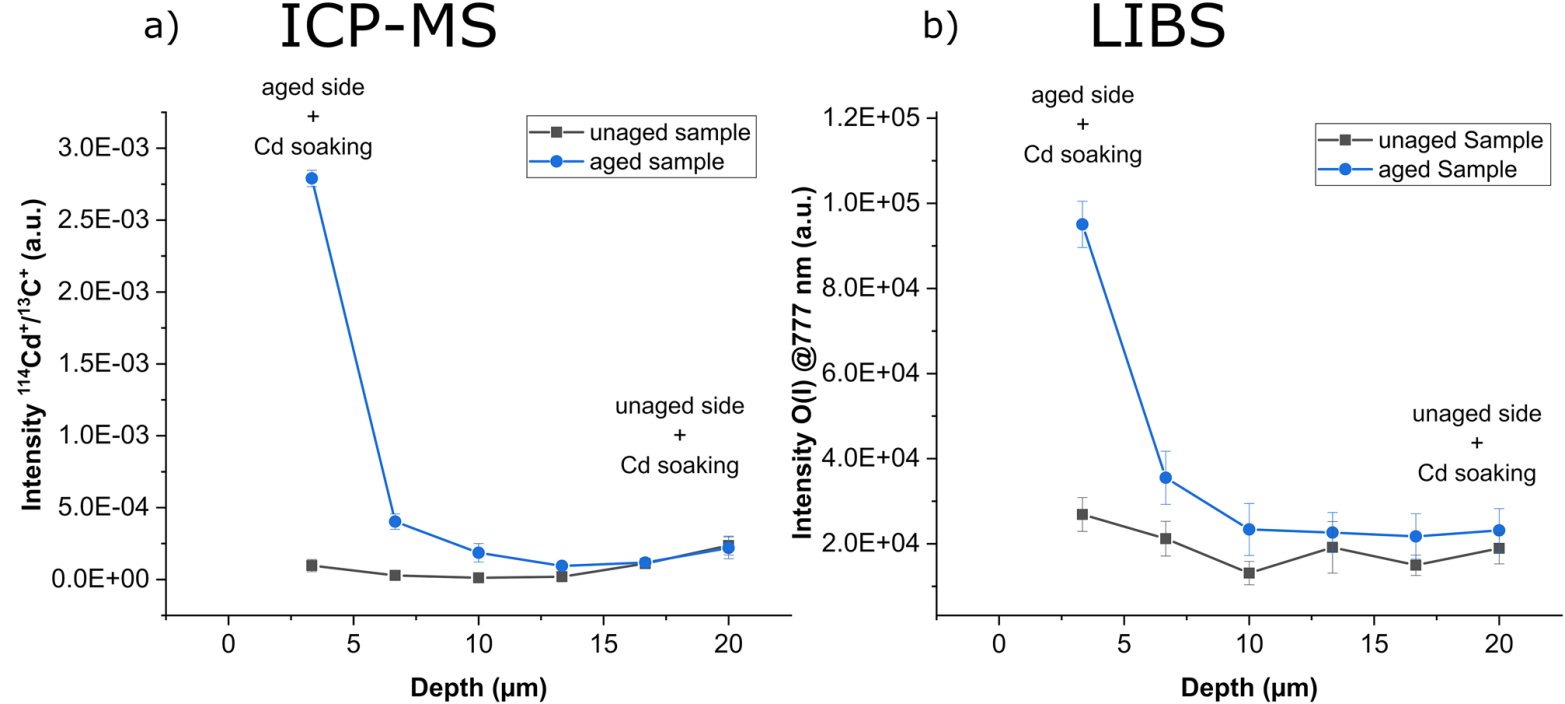
Depth profiles of an aged and an unaged polystyrene sample analyzed using a tandem LA-ICP-MS/LIBS setup: (**a**) shows the obtained depth profile of ^114^Cd detected with ICP-MS and (**b**) shows uptake of O detected with LIBS indicating oxidation of the sample.

Depth profiles from simultaneous analysis of Cd using ICP-MS and oxygen using LIBS are shown in Fig. 5. Compared to the unaged sample ICP-MS depth profiles reveal an increase of Cd at the aged side of the polystyrene film as well as a Cd diffusion gradient into the bulk of the aged polystyrene film. The unaged sample, which was not exposed to H_2_O_2_/UV radiation, shows only a small uptake of Cd. The unaged side of the aged sample shows the same uptake of Cd as the unaged sample. LIBS results reveal oxidation not only at the surface of the aged side of the aged sample but also 5 µm into depth. The opposite side does not show a significant increase of oxygen signal.

Summing up, the proposed tandem approach delivers the desired benefits in the characterization of degraded polymers. Nevertheless, it should be mentioned that laser systems that are usually employed for LIBS analysis are typically operated with higher laser fluence and longer wavelength (266 nm in this work). Compared to LA-ICP-MS, where a laser system operating at a wavelength of 213 nm was used this leads to more material being ablated with each laser shot, resulting in a decrease of the obtained depth resolution.

## Conclusion

In this work, laser based analytical techniques, namely LA-ICP-MS and LIBS were employed to investigate their applicability to study the behavior of various polymeric samples exposed to corrosive and degrading conditions. In contrast to techniques traditionally used for polymer characterization, LA-ICP-MS and LIBS offer trace element analysis as well as depth profiling. Additionally, LIBS inherently enables the detection of oxygen which can be used to detect oxidation of polymeric samples and also offers polymer specific signals which can be used to detect polymer degradation. These advantages were applied to 3 different examples covering a wide range of polymer applications demonstrating the beneficial information these laser-based techniques have to offer. Depth profiling of sulfur uptake in high-performance polyimides was investigated using LA-ICP-MS. Degradation of polymeric pigment binder mixtures from the field of cultural heritage science was analyzed using LIBS.

Besides conventional LA-ICP-MS and LIBS depth profiling, a tandem approach is also evaluated where signals of both techniques are acquired simultaneously. The obtained results from the tandem LA-ICP-MS/LIBS analysis show that the employment of such a measurement setup is beneficial to study the behavior of polymers under corrosive and degrading conditions. The measured uptake of the toxic trace metal Cd could be directly correlated with the information obtained for degradation and oxidation of the polymer sample.

Although the qualitative findings obtained in this feasibility study offer new and valuable information, future work will be focused on the collection of quantitative data to further improve existing knowledge about polymer degradation. Moreover, ongoing research will be devoted to enhancing the depth resolution of LIBS and tandem LA-ICP-MS/LIBS measurements, which is a precondition for the investigation of thin polymer films.

## Data Availability

The datasets generated during and/or analyzed during the current study are available from the corresponding author on reasonable request.
